# Supplementary material for: Detection of DNA base modifications by deep recurrent neural network on Oxford Nanopore sequencing data
Source: Nat Commun. 2019 Jun 4;10:2449. doi: 10.1038/s41467-019-10168-2 (PMC6547721; doi:10.1038/s41467-019-10168-2)
Supplement: Supplementary file 1 — Supplementary Information [file 41467_2019_10168_MOESM1_ESM.pdf]

Supplementary Information for

Detection of DNA base modifications by deep recurrent neural network on Oxford Nanopore sequencing data

Liu et al.

This supplementary file includes four sections: the detailed description of Oxford Nanopore sequencing datasets that we downloaded from previous publications (note that data generation for *Chlamydomonas reinhardtii* and HX1 were described in main texts), how to construct data for training/evaluation/testing of DeepMod (in particular modification labelling), followed by supplementary figures and tables for performance evaluation on different datasets.

Supplementary Notes

In this section, two groups of E. coli Nanopore data and one human Nanopore data were introduced. Please note that these datasets were published by existing works [1, 2, 3], and the introduction here was for your quick reference.

E. coli Nanopore sequencing data sequenced by Simpson et. al.* [1]*: This first group of E. coli Nanopore sequencing dataset contains both positive control data and negative control data of *E. coli*, and were sequenced by Simpson *et. al.* using Nanopore R9 sequencing techniques[1]. In their work[1], *E. coli* DNA was amplified using polymerase chain reaction (PCR), and then half of the PCR product were sequenced using Nanopore long-read techniques, and was considered as the negative control data. Thus, in negative control, there is no modification. The other half of the PCR product were treated with an enzyme of M.SssI methyltransferase in order to convert the majority of cytosines (C) in CpG contexts to 5-methylcytosine (5-mC) before being sequenced using the same Nanopore techniques. In this Nanopore sequencing dataset, there were 69,899 long reads for positive control and 111,238 long reads for negative control, and it is expected that almost all cytosines at CpG sites in the positive control (denoted as CG_MsssI for short) were assumed to be completely methylated by the enzyme, and all nucleotides include CpG sites in the negative control (denoted as UMR for short) were assumed to be completely unmethylated.

E. coli Nanopore sequencing data sequenced by Stoiber et. al.* [2]*: The second Nanopore sequencing dataset of E. coli were published by Stoiber et. al. [2]. This Nanopore dataset contains two negative control without any modification (denoted as Con1 and Con2 for short), and six positive control with two types of synthetically introduced methylations, 5mC and 6mA. Similar to the Nanopore sequencing dataset sequenced by Simpson et. al.[1], synthetically introduced methylations were treated by different enzymes after PCR amplification[2]. For 5mC, three methylases (M.Hhal, M.Mpel and M.Sssl) were used separately: M.Hhal is for GCGC motifs and the last two enzymes for CG motif (underlined C indicates the nucleotide in motifs to be methylased), and the three positive control were denoted as GCGC_Hhal, CG_MpeI and CG_SssI for short. Similarly, for 6mA, M.Taql was used to convert adenine in TCGA motif to 6mA (denoted as tcgA_TaqI for short), M.EcoRI to convert the second adenine in GAATTC motif to 6mA (denoted as gaAttc_EcoRI for short), and M.dam to convert adenine in GATC motif to 6mA (denoted as gAtc_dam for short).

In this dataset, the three 6mA data have 16,249 long reads in tcgA_TaqI, 17,557 long reads in gAtc_dam and 16,661 long reads in gaAttc_EcoRI, and the three 5mC data have 8,679 long reads for CG_SssI, 23,593 long reads for CG_MpeI and 28,168 long reads for GCGC_Hhal, while the two negative control have 23,762 and 34,170 long reads. For more detail, please refer to [2].

Human Nanopore sequencing dataset of NA12878: The human genome NA12878 has been well studied with different types of sequencing data including bisulfite sequencing data[4] and Nanopore long-read data[3]. Nanopore long-read data were generated by Jain *et al.* mainly using Nanopore R9.4 with ~30X coverage[3]. There was no PCR amplification before sequencing, and thus this Nanopore data contains native modifications in NA12878.

To evaluate DeepMod on NA12878 Nanopore sequencing data, the ground truth of methylation was obtained from bisulfite sequencing[4] with two replicates. The analysis of bisulfite sequencing provided percentages of methylations for cytosines in several motifs (CpG, CHG and CHH) together with coverage information, and the majority of methylations are for cytosines in CpG sites. The heterogeneity of sequenced cells could not be evaluated here.

Supplementary Methods

In this section, how to extract precise modification labels were introduced for synthetically introduced modifications and modifications detected by bisulfite sequencing. These labels were used for evaluating of DeepMod only, and not required and unknown in real-world applications.

Precise modification labels of events are necessary to build a DeepMod model in training process and to evaluate prediction performance of DeepMod in testing process. Reliable modification labelling of events is critical to build a well-trained model: if many labels are incorrect, the learning process would be incorrect. Nanopore sequencing data with synthetically introduced modifications and native modifications have different modification information, and thus were treated separately. Given a set of FAST5 files of Nanopore sequencing as input of DeepMod, the following procedures are used to extract precise modification labels of events in Nanopore long reads. Please note that the modification labeling here was only for training/testing process of DeepMod.

On synthetically introduced methylation data*:* For Nanopore sequencing with synthetical treatment, the molecules to be sequenced usually contains completely modified or completely un-modified bases for a nucleotide in a motif of interest. Since the synthetically introduced methylations are usually motif-based (for example, in CG_MSssI, almost all cytosine in CpG sites were methylated, and none of other bases should be modified), each event in negative control Nanopore sequencing data were generated from un-methylated bases. In a synthetically introduced methylation dataset, methylation and un-methylation labeling was a little more complicated, because of error rate of Nanopore sequencing, the slight difference between sequenced reads and their reference genome and other factors. Thus the following procedures were used for methylation and un-methylation labeling for a Nanopore sequencing data with synthetically introduced methylations based on motifs.

First, a sequence of bases from events were extracted from each a long read, and the sequence was aligned with a reference genome (*E. coli* strand K-12 sub-strand MG1655[5] for E. coli datasets and hg38 for human Nanopore sequencing data). Then according to the alignment, we found out reliable motif-based methylations which were synthetically introduced by methylases. In detail, given a motif, for example, CpG sites, C in a CpG site was labelled to be methylated if (i) there were not more than 2 gaps in a 7-base window centered at that C, or (ii) there were not more than 3 gaps in a 13-base window centered at that C. The maximum number of gaps in a small region was used to eliminate poor alignment with more gaps, and CpG sites in poor alignment regions were not used. Meanwhile, 13-base

window is used to avoid the incorrect small-region alignment due to methylations (methylation might results in wrong basecalling in a k-mer such as 5-mer) rather than due to alignment errors, while 7-base window is used to avoid other effect on the alignment. 7 is the minimum odd number larger than 5 and 5-mer is usually used for event in fast5 files, while 13 is almost twice of 7. These numbers is selected based on our understanding and not optimal.

After that, all other bases in a long read would have an un-methylation label, but one adjacent upstream and downstream events of the C in any CpG would be excluded from training and testing data without any methylation or un-methylation label. That is, long reads with synthetically introduced methylations contain methylated events, unmethylated events, and other events which were not used for training/testing. For other types of motifs, the similar procedures could be used to label events in long reads with synthetically introduced methylations.

Usually, a given reference genome contains a roughly known number of a certain motifs. For example, in a reference *E. coli* genome, there are 693,586 CpG sites among ~4.64Mb nucleotides. The information for other motifs could also be found in [2].

On real native data with bisulfite sequencing: In Nanopore sequencing data with native DNA molecules, heterogeneous cells were usually sequenced, and then each strand-sensitive genome position was associated with a unknown methylation percentage rather than complete methylation (the methylation percent is 100%) or complete un-methylation (the methylation percent is 0%). Usually, bisulfite sequencing was used as a gold standard of DNA methylation. Thus, for a genome with both Nanopore sequencing data and bisulfite sequencing data, the criteria below was used to obtain high-quality methylation and un-methylation labels for events in long reads: on one hand, if a cytosine at a particular genomic position in a reference genome (hg38) has both >90% of methylations in two replicates of bisulfite sequencing, a cytosine at the corresponding position from long reads was considered to be completely methylated when that cytosine from long reads was just aligned with the cytosine at the particular genomic position in hg38; on other hand, if a nucleotide at a particular genomic position in hg38 has 0% methylations in both replicates of bisulfite sequencing, a cytosine at the corresponding position from long reads was considered to be completely un-methylated when that cytosine from long reads was just aligned with the cytosine at the particular genomic position in hg38. Nucleotides at certain positions with methylation percent between 0% and 90% were not considered in this testing process. The number of completely methylated/un-methylated cytosines from NA12878 (HX1) was given in Supplementary Table 4 (Supplementary Table 5).

Supplementary Discussion

Evaluation on Escherichia coli data sequenced by Simpson et. al. [1]: This Nanopore dataset of *E. coli* has high-coverage, and thus it was used to see how different hyper-parameters affect DeepMod performance in modification prediction. To overcome certain overfitting issues, two independent validation strategies were used to evaluate DeepMod on this data. One strategy is read-based, where all long reads were divided into two groups: one group, with 90% long reads in CG_MSssI (positive control) and 90% long reads in UMR (negative control), was used to train DeepMod, and the remained 10% long reads (10% long reads in CG_MSssI and 10% long reads in UMR) were used for testing DeepMod. Under

this validation strategy, long reads in testing groups and in training groups might be aligned with same reference positions.

Thus, the second validation strategy is region-based independent validation, where all long reads or some bases of long reads mapped to the genomic positions from 1,000,000 to 2,000,000 of *E. coli* were used for testing, no matter long reads were from CG_MSssI or UMR; the rest of long reads were used for training DeepMod.

To train DeepMod, there is a hyper-parameter of window size, w. No prior knowledge can guarantee which w is best. To select a better w, we ranged w from 7, to 11, to 15, to 21, to 31, and then to 51 with a step of 10. The per-call validation performance with different ws and the two validation strategies were shown in **Error! Reference source not found.** together with 57-feature or 7-feature description and the number of times long reads in UMR were used for training. The per-call performance was evaluated by precision, recall, accuracy and F1-score. It can be seen from **Error! Reference source not found.** that 57-feature description provided similar performance to 7-feature description did under the two validation strategies. Thus, 7-feature description was used by default in DeepMod.

Meanwhile from **Error! Reference source not found.**, when w increases, F1-score also increases, although the increase of F1-score become smaller. However, with a larger w, more resources (time, CPU and memory) were needed for both training and prediction. In real applications, w=21 was selected as default setting. $w = 21$ was used on other Nanopore datasets without further optimization. This value of w was larger than 5 or 6, but this did not mean that all adjacent w bases of a base or as far as w bases of a base would affect signals of an event associated with the base. However, if users have enough resources and time, we recommended a larger w with better performance.

Supplementary Tables

Supplementary Table 1. Per-call performance of DeepMod with independent validation on *E. coli* nanopore data of CG_MSssI (a nanopore data of PCR-amplified and enzyme-treated reads where almost CpG sites were methylated) and of UMR (a nanopore data of PCR-amplified reads where no modification would be available). w is the window size. Read-based validation (in *italic*) means 10% reads were used for testing while 90% reads were used for training DeepMod, no matter reads were from CG_MSssI or UMR, while Region-based validation means reads or bases mapped to the genomic positions from 1,000,000 to 2,000,000 were used for testing and reads/bases mapped to other genomic positions were used to train DeepMod. 57-feature description represents 50 count values of 50 bins and mean, standard deviation and length for an event together with base information, while 7-feature description has only three summarization features together with base information. r1, r2, r3 and r4 indicates that how many times (1, 2, 3 and 4) long reads in UMR were used for DeepMod. The descriptions of different metrics were described in Methods. MCC is Matthews correlation coefficient.

w	Metrics	*Read-based validation*								Region-based validation							
		57-feature description				*7-feature description*				57-feature description				7-feature description			
		r1	*r2*	*r3*	*r4*	*r1*	*r2*	*r3*	*r4*	r1	r2	r3	r4	r1	r2	r3	r4
7	Precision	*0.780*	*0.788*	*0.790*	*0.777*	**0.762**	**0.774**	**0.776**	**0.771**	0.777	0.777	0.785	0.798	**0.751**	**0.758**	**0.758**	**0.768**
	Recall	*0.837*	*0.835*	*0.834*	*0.848*	**0.829**	**0.827**	**0.826**	**0.834**	0.840	0.843	0.839	0.827	**0.834**	**0.840**	**0.841**	**0.835**
	F1-score	*0.808*	*0.811*	*0.812*	*0.811*	**0.794**	**0.800**	**0.800**	**0.801**	0.808	0.809	0.811	0.812	**0.790**	**0.797**	**0.798**	**0.800**
	Accuracy	*0.955*	*0.956*	*0.956*	*0.955*	**0.951**	**0.953**	**0.953**	**0.953**	0.956	0.956	0.957	0.958	**0.951**	**0.953**	**0.953**	**0.954**
	MCC	*0.783*	*0.787*	*0.788*	*0.787*	**0.767**	**0.774**	**0.774**	**0.775**	0.783	0.785	0.787	0.788	**0.764**	**0.772**	**0.772**	**0.775**
11	Precision	*0.824*	*0.828*	*0.843*	*0.816*	**0.807**	**0.826**	**0.822**	**0.825**	0.823	0.821	0.829	0.844	**0.799**	**0.810**	**0.812**	**0.820**
	Recall	*0.875*	*0.881*	*0.870*	*0.893*	**0.869**	**0.864**	**0.871**	**0.870**	0.876	0.882	0.880	0.867	**0.866**	**0.873**	**0.874**	**0.869**
	F1-score	*0.849*	*0.854*	*0.857*	*0.853*	**0.837**	**0.845**	**0.846**	**0.847**	0.848	0.850	0.853	0.856	**0.831**	**0.840**	**0.842**	**0.844**
	Accuracy	*0.965*	*0.966*	*0.967*	*0.965*	**0.962**	**0.964**	**0.964**	**0.964**	0.965	0.966	0.967	0.968	**0.961**	**0.963**	**0.964**	**0.964**
	MCC	*0.829*	*0.835*	*0.838*	*0.834*	**0.816**	**0.825**	**0.826**	**0.827**	0.829	0.832	0.835	0.837	**0.810**	**0.820**	**0.822**	**0.824**
15	Precision	*0.850*	*0.847*	*0.867*	*0.831*	**0.833**	**0.852**	**0.845**	**0.853**	0.850	0.842	0.847	0.861	**0.830**	**0.832**	**0.833**	**0.844**
	Recall	*0.891*	*0.901*	*0.889*	*0.915*	**0.886**	**0.882**	**0.892**	**0.888**	0.886	0.899	0.899	0.888	**0.879**	**0.893**	**0.894**	**0.888**
	F1-score	*0.870*	*0.873*	*0.878*	*0.871*	**0.859**	**0.867**	**0.868**	**0.870**	0.868	0.870	0.872	0.875	**0.854**	**0.861**	**0.863**	**0.865**
	Accuracy	*0.970*	*0.971*	*0.972*	*0.969*	**0.967**	**0.969**	**0.969**	**0.970**	0.970	0.970	0.971	0.972	**0.967**	**0.968**	**0.968**	**0.969**
	MCC	*0.853*	*0.857*	*0.862*	*0.855*	**0.841**	**0.849**	**0.851**	**0.854**	0.851	0.853	0.856	0.859	**0.835**	**0.844**	**0.845**	**0.849**
21	Precision	*0.877*	*0.872*	*0.893*	*0.851*	**0.867**	**0.878**	**0.873**	**0.883**	0.879	0.871	0.872	0.886	**0.860**	**0.857**	**0.864**	**0.876**
	Recall	*0.908*	*0.920*	*0.907*	*0.935*	**0.903**	**0.906**	**0.912**	**0.908**	0.902	0.915	0.917	0.908	**0.897**	**0.914**	**0.912**	**0.904**
	F1-score	*0.892*	*0.896*	*0.900*	*0.891*	**0.885**	**0.892**	**0.892**	**0.895**	0.890	0.893	0.894	0.897	**0.878**	**0.884**	**0.887**	**0.890**
	Accuracy	*0.975*	*0.976*	*0.977*	*0.974*	**0.973**	**0.975**	**0.975**	**0.976**	0.975	0.976	0.976	0.977	**0.972**	**0.974**	**0.974**	**0.975**
	MCC	*0.878*	*0.882*	*0.887*	*0.878*	**0.870**	**0.878**	**0.878**	**0.882**	0.876	0.879	0.881	0.884	**0.863**	**0.870**	**0.873**	**0.876**
31	Precision	*0.911*	*0.904*	*0.923*	*0.882*	**0.899**	**0.910**	**0.903**	**0.913**	0.911	0.900	0.905	0.913	**0.900**	**0.894**	**0.897**	**0.906**
	Recall	*0.925*	*0.939*	*0.928*	*0.952*	**0.927**	**0.927**	**0.936**	**0.930**	0.921	0.935	0.934	0.929	**0.914**	**0.931**	**0.932**	**0.926**
	F1-score	*0.918*	*0.921*	*0.925*	*0.916*	**0.913**	**0.918**	**0.919**	**0.921**	0.916	0.917	0.919	0.921	**0.907**	**0.912**	**0.914**	**0.916**
	Accuracy	*0.981*	*0.982*	*0.983*	*0.980*	**0.980**	**0.981**	**0.981**	**0.982**	0.981	0.981	0.982	0.982	**0.979**	**0.980**	**0.981**	**0.981**
	MCC	*0.907*	*0.911*	*0.916*	*0.905*	**0.902**	**0.908**	**0.908**	**0.911**	0.906	0.907	0.909	0.911	**0.895**	**0.901**	**0.904**	**0.905**
41	Precision	*0.931*	*0.923*	*0.941*	*0.898*	**0.924**	**0.932**	**0.926**	**0.932**	0.933	0.917	0.921	0.928	**0.919**	**0.917**	**0.919**	**0.930**
	Recall	*0.938*	*0.952*	*0.940*	*0.963*	**0.938**	**0.940**	**0.947**	**0.945**	0.932	0.949	0.948	0.945	**0.929**	**0.945**	**0.945**	**0.938**
	F1-score	*0.935*	*0.937*	*0.941*	*0.929*	**0.931**	**0.936**	**0.936**	**0.938**	0.932	0.933	0.934	0.937	**0.924**	**0.930**	**0.931**	**0.934**
	Accuracy	*0.985*	*0.986*	*0.987*	*0.983*	**0.984**	**0.985**	**0.985**	**0.986**	0.985	0.985	0.985	0.986	**0.983**	**0.984**	**0.985**	**0.985**
	MCC	*0.926*	*0.929*	*0.933*	*0.921*	**0.922**	**0.928**	**0.928**	**0.930**	0.924	0.924	0.926	0.929	**0.915**	**0.922**	**0.923**	**0.925**
51	Precision	*0.945*	*0.937*	*0.953*	*0.913*	**0.938**	**0.944**	**0.940**	**0.944**	0.943	0.931	0.933	0.942	**0.936**	**0.929**	**0.935**	**0.943**
	Recall	*0.946*	*0.959*	*0.948*	*0.969*	**0.946**	**0.950**	**0.955**	**0.954**	0.943	0.957	0.957	0.953	**0.939**	**0.953**	**0.952**	**0.947**
	F1-score	*0.946*	*0.948*	*0.950*	*0.940*	**0.942**	**0.947**	**0.947**	**0.949**	0.943	0.944	0.945	0.948	**0.938**	**0.941**	**0.943**	**0.945**
	Accuracy	*0.988*	*0.988*	*0.989*	*0.986*	**0.987**	**0.988**	**0.988**	**0.988**	0.987	0.987	0.988	0.988	**0.986**	**0.987**	**0.987**	**0.988**
	MCC	*0.939*	*0.941*	*0.944*	*0.933*	**0.935**	**0.940**	**0.940**	**0.942**	0.936	0.937	0.938	0.941	**0.930**	**0.934**	**0.936**	**0.938**

Supplementary Table 2. Cross-validation per-call performance of DeepMod with independent validation on *E. coli* nanopore data of CG_MSssI (a nanopore data of PCR-amplified and enzyme-treated reads where almost CpG sites were methylated) and of UMR (a nanopore data of PCR-amplified reads where no modification would be available). Tested regions means the region in the row were used for testing and other genomic positions were used to train DeepMod. r1, r2, r3 and r4 indicates that how many times (1, 2, 3 and 4) long reads in UMR were used for DeepMod. The descriptions of different metrics were described in Methods. MCC is Matthews correlation coefficient.

Tested regions	Metrics	*r1*	*r2*	*r3*	*r4*
[0, 1000000]	Precision	0.859	0.872	0.868	0.879
	Recall	0.904	0.906	0.912	0.906
	F1-score	0.881	0.889	0.889	0.892
	Accuracy	0.972	0.974	0.974	0.975
	MCC	0.866	0.874	0.875	0.878
[1000000, 2000000]	Precision	0.860	0.857	0.864	0.876
	Recall	0.897	0.914	0.912	0.904
	F1-score	0.878	0.884	0.887	0.890
	Accuracy	0.972	0.974	0.974	0.975
	MCC	0.863	0.870	0.873	0.876
[2000000, 3000000]	Precision	0.848	0.866	0.869	0.874
	Recall	0.912	0.909	0.911	0.910
	F1-score	0.879	0.887	0.889	0.892
	Accuracy	0.972	0.974	0.974	0.975
	MCC	0.864	0.872	0.875	0.878
[3000000, 4000000]	Precision	0.862	0.868	0.881	0.859
	Recall	0.906	0.912	0.907	0.923
	F1-score	0.884	0.889	0.894	0.890
	Accuracy	0.972	0.974	0.975	0.974
	MCC	0.868	0.875	0.880	0.876
[4000000, 4700000]	Precision	0.848	0.872	0.859	0.887
	Recall	0.908	0.909	0.921	0.904
	F1-score	0.877	0.890	0.889	0.895
	Accuracy	0.971	0.974	0.974	0.976
	MCC	0.861	0.876	0.875	0.881

Supplementary Table 3. Confusion matrix for DeepMod on E. coli data using a cutoff of methylation percentage 0.1. Pos: methylation in ground truth or methylation prediction; Neg: non-methylation in ground truth or non-methylation prediction. The first column is about the motif followed by an enzyme to methylate bases whose name is capital in motifs.

	coverage>=1						coverage>=5			
			Ground truth					Ground truth		
			Pos	*Neg*	*Total*			*Pos*	*Neg*	*Total*
**Cg_MpeI**	*Prediction*	*Pos*	609,531	120,348	729,879	*Prediction*	*Pos*	590,771	120,272	711,043
		Neg	83,987	573,079	657,066		*Neg*	75,678	572,591	648,269
		Total	693,518	693,427	1,386,945		*Total*	666,449	692,863	1,359,312
**Cg_SssI**			*Pos*	*Neg*	*Total*			*Pos*	*Neg*	*Total*
	Prediction	*Pos*	673,204	120,348	793,552	*Prediction*	*Pos*	338,411	120,272	458,683
		Neg	9,322	573,079	582,401		*Neg*	136	572,591	572,727
		Total	682,526	693,427	1,375,953		*Total*	338,547	692,863	1,031,410
**gCgc_HhaI**			*Pos*	*Neg*	*Total*			*Pos*	*Neg*	*Total*
	Prediction	*Pos*	68,054	3,973	72,027	*Prediction*	*Pos*	67,331	3,965	71,296
		Neg	2,118	66,187	68,305		*Neg*	2,040	66,129	68,169
		Total	70,172	70,160	140,332		*Total*	69,371	70,094	139,465
**gaAttc_EcoRI**			*Pos*	*Neg*	*Total*			*Pos*	*Neg*	*Total*
	Prediction	*Pos*	204	0	204	*Prediction*	*Pos*	153	0	153
		Neg	73	280	353		*Neg*	41	280	321
		Total	277	280	557		*Total*	194	280	474
**gAtc_dam**			*Pos*	*Neg*	*Total*			*Pos*	*Neg*	*Total*
	Prediction	*Pos*	7,102	2,179	9,281	*Prediction*	*Pos*	6,455	2,179	8,634
		Neg	714	5,652	6,366		*Neg*	492	5,641	6,133
		Total	7,816	7,831	15,647		*Total*	6,947	7,820	14,767
**tcgA_TaqI**			*Pos*	*Neg*	*Total*			*Pos*	*Neg*	*Total*
	Prediction	*Pos*	4,729	506	5,235	*Prediction*	*Pos*	3,073	506	3,579
		Neg	1,622	5,897	7,519		*Neg*	583	5,891	6,474
		Total	6,351	6,403	12,754		*Total*	3,656	6,397	10,053

Supplementary Table 4. The number of completely methylated (Meth) and completely un-methylated (Un-meth) cytosines in CpG sites in NA12878. Completely methylated cytosine are those cytosines at particular genomic positions which have >90% methylation percentage for both replicates in bisulfite sequencing of NA12878 with coverage requirements in corresponding columns, while completely un-methylated cytosine are those cytosines at particular genomic positions which have =0% methylation percentage for both replicates in bisulfite sequencing of NA12878. Prec is the precision and Rec is recall for a binary classification with a threshold of predicted methylation percentage=0.5.

	**Coverage>=1**			**Coverage>=5**				**Coverage>=10**	
	**Not used**	**Unmeth**	**Meth**	**Unmeth**	**Meth**	**Prec**	**Rec**	**Unmeth**	**Meth**
**chr1**	2,871,679	670,100	1,055,298	467,895	882,473	0.986	0.976	278,266	618,841
**chr2**	2,787,980	602,897	903,266	450,601	772,309	0.984	0.974	295,630	566,033
**chr3**	2,120,764	453,635	703,000	348,478	603,957	0.987	0.978	233,460	444,686
**chr4**	1,987,331	438,488	511,922	347,394	431,662	0.983	0.979	246,785	317,202
**chr5**	1,960,268	408,746	602,031	315,268	511,163	0.987	0.980	212,473	376,541
**chr6**	1,911,328	414,387	640,591	315,975	549,948	0.989	0.981	210,694	408,094
**chr7**	2,017,645	407,168	697,757	295,251	571,115	0.989	0.979	187,221	392,520
**chr8**	1,729,141	379,701	476,602	287,469	401,463	0.987	0.981	192,793	288,231
**chr9**	1,529,082	347,567	508,289	245,431	420,893	0.989	0.981	150,257	290,352
**chr10**	1,755,015	381,937	556,222	283,495	472,335	0.988	0.981	178,142	338,518
**chr11**	1,675,066	422,129	517,249	303,378	430,250	0.987	0.980	187,648	297,015
**chr12**	1,611,621	351,343	620,985	257,146	523,111	0.991	0.981	162,073	370,010
**chr13**	1,119,063	233,494	308,470	182,642	260,296	0.987	0.982	129,292	191,023
**chr14**	1,049,167	243,650	402,780	178,168	342,046	0.991	0.981	112,569	245,063
**chr15**	1,049,963	236,800	406,375	168,192	345,013	0.990	0.981	101,797	248,574
**chr16**	1,303,836	304,356	550,850	206,181	444,933	0.990	0.979	115,123	282,439
**chr17**	1,349,311	337,038	637,899	211,214	522,645	0.991	0.979	104,028	338,774
**chr18**	976,873	206,334	247,794	157,219	206,978	0.985	0.982	109,482	150,073
**chr19**	1,146,244	319,910	603,083	195,989	457,358	0.991	0.978	86,288	253,178
**chr20**	963,565	221,177	314,429	154,669	259,434	0.988	0.978	90,907	174,100
**chr21**	502,296	103,630	146,057	76,170	117,929	0.986	0.980	49,838	78,880
**chrX**	1,901,019	306,943	229,057	254,964	174,105	0.968	0.979	190,395	113,264

Supplementary Table 5. The number of completely methylated (Meth) and completely un-methylated (Un-meth) cytosines in CpG sites in HX1. Completely methylated cytosine are those cytosines at particular genomic positions which have >90% methylation percentage for both replicates in bisulfite sequencing of HX1 with coverage requirements in corresponding columns, while completely un-methylated cytosine are those cytosines at particular genomic positions which have =0% methylation percentage for both replicates in bisulfite sequencing of HX1. Prec is the precision and Rec is recall for a binary classification with a threshold of predicted methylation percentage=0.5.

	**Coverage>=3**			
	**Un-meth**	**Meth**	**Prec**	**Rec**
**chr1**	142,626	1,291,541	0.990	0.983
**chr2**	114,997	1,265,532	0.991	0.986
**chr3**	88,550	978,097	0.991	0.988
**chr4**	73,850	828,524	0.990	0.988
**chr5**	78,912	858,739	0.991	0.987
**chr6**	94,942	871,455	0.989	0.988
**chr7**	78,161	873,128	0.991	0.985
**chr8**	63,004	731,614	0.991	0.986
**chr9**	63,314	647,986	0.991	0.984
**chr10**	72,274	771,784	0.991	0.984
**chr11**	78,275	684,732	0.990	0.982
**chr12**	78,622	743,997	0.990	0.986
**chr13**	41,564	472,591	0.990	0.987
**chr14**	52,448	484,153	0.990	0.985
**chr15**	50,450	487,773	0.991	0.985
**chr16**	57,980	559,559	0.991	0.980
**chr17**	78,275	615,130	0.990	0.981
**chr18**	32,397	393,366	0.991	0.986
**chr19**	80,271	498,454	0.989	0.977
**chr20**	40,756	389,648	0.991	0.981
**chr21**	17,384	200,525	0.991	0.981
**chr22**	30,343	292,362	0.991	0.976
**chrX**	24,557	370,359	0.987	0.985
**chrY**	1,338	30,825	0.989	0.967

Supplementary Figures

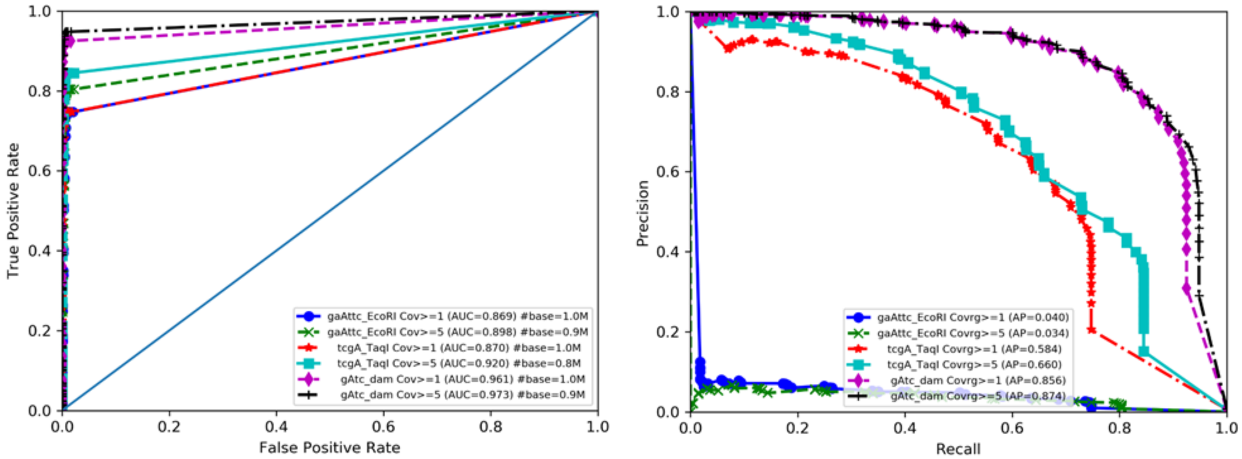

(a) AUC of 6mA prediction on E. coli ***(b) AP of 6mA prediction on E. coli***

Supplementary Figure 1. Performance evaluation of DeepMod on E. Coli. (a, b) AP and AUC plots for 6mA prediction of DeepMod on E. coli for three synthetically introduced 6mA by EcoRI (gaAttc_EcoRI for GAATTC motif), TaqI (tcgA_TaqI for TCGA motif) and dam (gAtc_dam for GATC motif) respectively. The evaluation was conducted by comparing modification of 6mA against all adenines. Cov: coverage. #base: total number of bases used in the evaluation.

Supplementary References

1. Simpson JT, Workman RE, Zuzarte PC, David M, Dursi LJ, Timp W. Detecting DNA cytosine methylation using nanopore sequencing. *Nature methods* **14**, 407-410 (2017).

2. Stoiber MH*, et al.* De novo Identification of DNA Modifications Enabled by Genome-Guided Nanopore Signal Processing. *bioRxiv* **10.1101/094672**, (2017).

3. Jain M*, et al.* Nanopore sequencing and assembly of a human genome with ultra-long reads. *Nat Biotechnol* **36**, 338-345 (2018).

4. An integrated encyclopedia of DNA elements in the human genome. *Nature* **489**, 57-74 (2012).

5. Blattner FR*, et al.* The complete genome sequence of Escherichia coli K-12. *Science* **277**, 1453-1462 (1997).
